# Census Bulletin, No. 20

**Published:** 1891-03

**Authors:** 


### BOOK NOTICES.

CENSUS BULLETIN, NO. 20. Hon. Robert P. Porter, Superintendent of the Census.

This bulletin relates to the mining of anthracite coal, from which it appears that the total number of tons mined was 25,575,875, valued at $42,172,942.

